# The genome sequence of the Eurasian red squirrel, *Sciurus vulgaris* Linnaeus 1758

**DOI:** 10.12688/wellcomeopenres.15679.1

**Published:** 2020-02-03

**Authors:** Daniel Mead, Kathryn Fingland, Rachel Cripps, Roberto Portela Miguez, Michelle Smith, Craig Corton, Karen Oliver, Jason Skelton, Emma Betteridge, Jale Dolucan, Olga Dudchenko, Arina D. Omer, David Weisz, Erez Lieberman Aiden, Olivier Fedrigo, Jacquelyn Mountcastle, Erich Jarvis, Shane A. McCarthy, Ying Sims, James Torrance, Alan Tracey, Kerstin Howe, Richard Challis, Richard Durbin, Mark Blaxter

**Affiliations:** 1 Tree of Life, Wellcome Sanger Institute, Cambridge, CB10 1SA, UK; 2 School of Animal, Rural and Environmental Sciences, Nottingham Trent University, Nottingham, NG25 0QF, UK; 3 The Wildlife Trust for Lancashire, Manchester and North Merseyside, Preston, PR5 6BY, UK; 4 Department of Life Sciences, Natural History Museum, London, SW7 5BD, UK; 5 Baylor College of Medicine, Houston, TX, 77030, USA; 6 Laboratory of Neurogenetics of Language, The Rockefeller University, New York, NY, 10065, USA; 7 Howard Hughes Medical Institute, Chevy Chase, MD, 20815, USA; 8 Department of Genetics, University of Cambridge, Cambridge, CB2 3EH, UK

**Keywords:** Sciurus vulgaris, red squirrel, genome sequence, chromosomal

## Abstract

We present a genome assembly from an individual male *Sciurus vulgaris* (the Eurasian red squirrel; Vertebrata; Mammalia; Eutheria; Rodentia; Sciuridae). The genome sequence is 2.88 gigabases in span. The majority of the assembly is scaffolded into 21 chromosomal-level scaffolds, with both X and Y sex chromosomes assembled.

## Species taxonomy

Eukaryota; Metazoa; Chordata; Craniata; Vertebrata; Euteleostomi; Mammalia; Eutheria; Euarchontoglires; Glires; Rodentia; Sciuromorpha; Sciuridae; Sciurinae; Sciurini; Sciurus; *Sciurus vulgaris* Linnaeus 1758 (NCBI txid 55149).

## Background

The Eurasian red squirrel, *Sciurus vulgaris*, is native to northern Eurasia. In the Atlantic Archipelago of Britain and Ireland, *S. vulgaris* is under threat from anthropogenic pressure on its native woodland habitats ^1^, and from competition from the introduced American grey squirrel, *Sciurus carolinensis*, particularly mediated by squirrelpox virus ( Chantrey *et al*., 2014). The current population of *S. vulgaris* in the Atlantic Archipelago is estimated to be 150,000, and there are extensive efforts to conserve this species and expand its range ( Hardouin *et al*., 2019). Here we present a chromosomally assembled genome sequence for *S. vulgaris*, based on a male specimen from Britain. This genome sequence will be of utility in population genomic analysis of fragmented *S. vulgaris* populations ( Barratt *et al.*, 1999), in managing reintroductions and in investigating the biology of susceptibility to squirrelpox virus ( Darby *et al*., 2014).

## Genome sequence report

**Figure 1.**
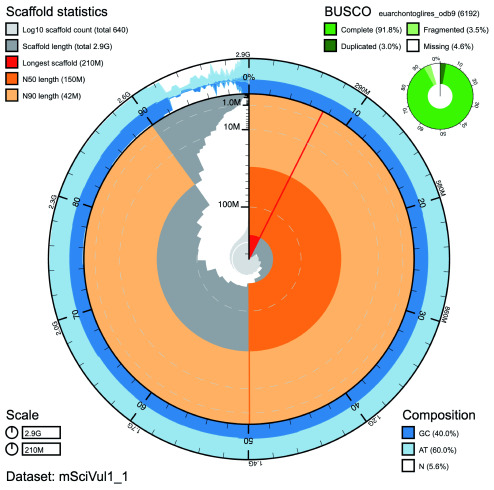
Genome assembly of Sciurus vulgaris mSciVul1: Metrics. BlobToolKit Snailplot showing N50 metrics for *S. vulgaris* assembly mSciVul1 and BUSCO scores for the Euarchontoglires set of orthologues. The interactive version of this figure is available here.

The genome was sequenced from DNA extracted from a from a naturally deceased male *S. vulgaris* collected as part of a squirrel monitoring project run by the Wildlife Trust for Lancashire, Manchester and North Merseyside. A total of 51-fold coverage in Pacific Biosciences single-molecule long reads (N50 19 kb) and 44-fold coverage in 10X Genomics read clouds (from molecules with an estimated N50 of 69 kb) were generated. Primary assembly contigs were scaffolded with 10X read clouds, chromosome conformation HiC data, and 111-fold coverage of Bionano optical maps. The final assembly has a total length of 2.88 Gb in 638 sequence scaffolds with a scaffold N50 of 153.9 Mb ( Table 1). The majority, 92.7%, of the assembly sequence was assigned to 21 chromosomal pseudomolecules representing 19 autosomes (numbered by sequence length), and the X and Y sex chromosomes ( Figure 1– Figure 4; Table 2). The assembly has a BUSCO ( Simão *et al.*, 2015) completeness of 93.8% using the mammalia_odb9 reference set. The primary assembly is a large-scale mosaic of both haplotypes (i.e. is not fully phased) and we have therefore also deposited the contigs corresponding to the alternate haplotype. The genome can be compared to that of the grey squirrel, *Sciurus carolinensis*, which we have also assembled.

**Table 1.** Genome data for *Sciurus vulgaris* mSciVul1.

Project accession data	
Assembly identifier	mSciVul1
Species	*Sciurus vulgaris*
Specimen	NHMUK ZD.2019.213
NCBI taxonomy ID	55149
BioProject	PRJEB35381
Biosample ID	SAMEA994733
Isolate information	Wild isolate; male
Raw data accessions	
PacificBiosciences SEQUEL I	ERR3147845-ERR3147850, ERR3151029, ERR3151031-ERR3151033, ERR3151038-ERR3151041, ERR3151043-ERR3151044, ERR3168377-ERR3168381, ERR3197128, ERR3197129, ERR3218392, ERR3284521, ERR3291651-ERR3291656, ERR3291658, ERR3291671-ERR3291674
10X Genomics Illumina	ERR3316125-ERR3316132
Hi-C Illumina	SRR10119465
BioNano data and assembly	ERZ1283748
Genome assembly	
Assembly accession	GCA_902686455
Accession of alternate haplotype	GCA_902685485
Span (Mb)	2,879
Number of contigs	1,799
Contig N50 length (Mb)	16.3
Number of scaffolds	638
Scaffold N50 length (Mb)	153.9
Longest scaffold (Mb)	213.2
BUSCO * genome score	C:93.8%[S:90.7%,D:3.1%],F:3.0%,M:3.2%,n:4104

* BUSCO scores based on the mammalia_odb9 BUSCO set using v3.0.2. C= complete [S= single copy, D=duplicated], F=fragmented, M=missing, n=number of orthologues in comparison. A full set of BUSCO scores is available at https://blobtoolkit.genomehubs.org/view/mSciVul1_1/dataset/mSciVul1_1/busco.

**Figure 2.**
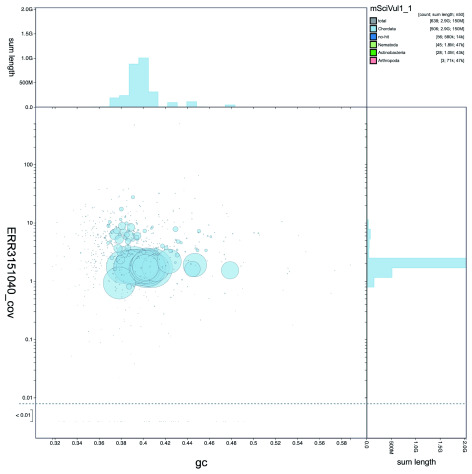
Genome assembly of Sciurus *vulgaris mSciVul1*: GC-coverage plot. BlobToolKit GC-coverage plot of *S. vulgaris* mSciVul1. The interactive version of this figure is available here.

**Figure 3.**
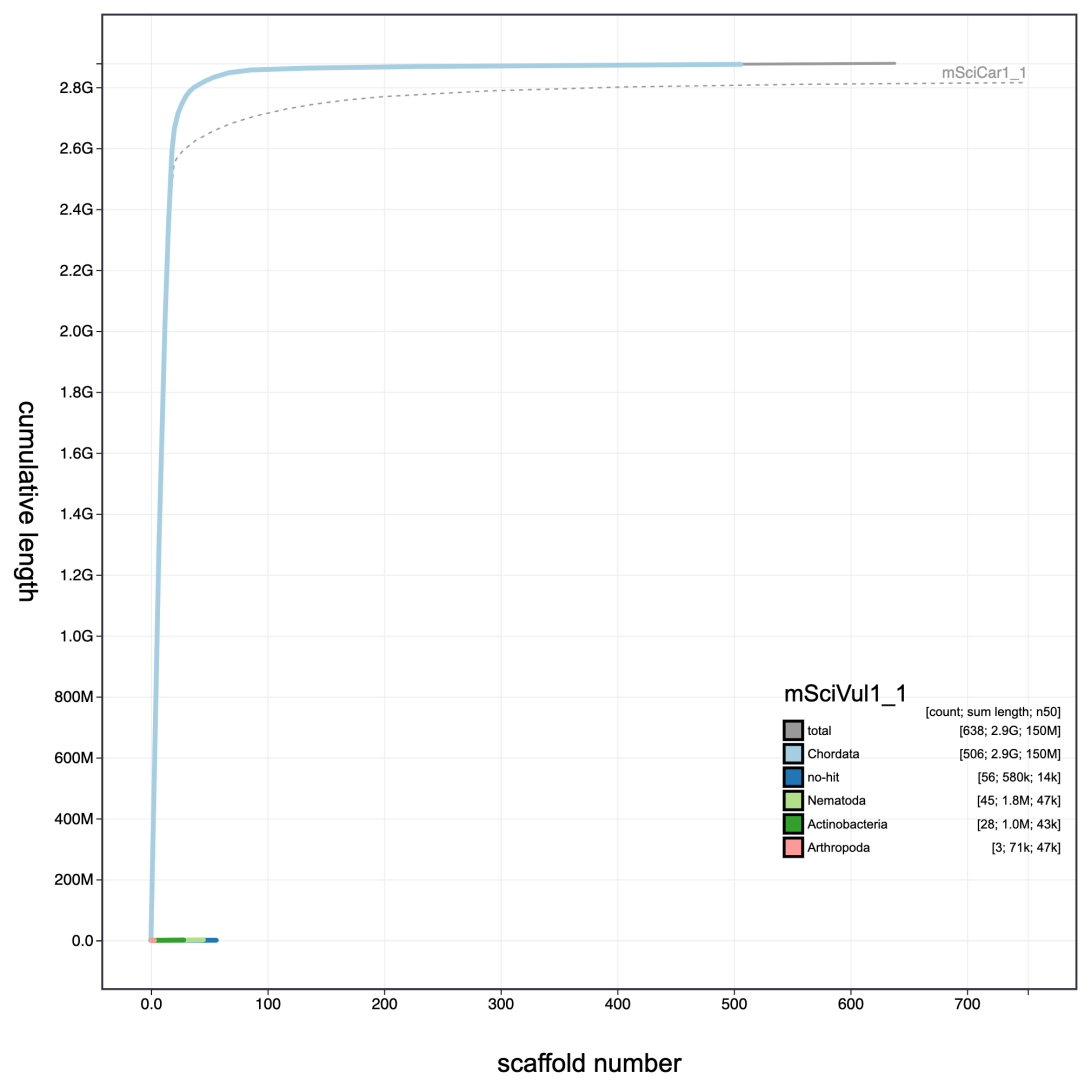
Genome assembly of *Sciurus vulgaris* mSciVul1: Cumulative sequence plot. Dashed line shows the cumulative sequence plot of *S. carolinensis* mSciCar1 for comparison. The interactive version of this figure is available here.

**Figure 4.**
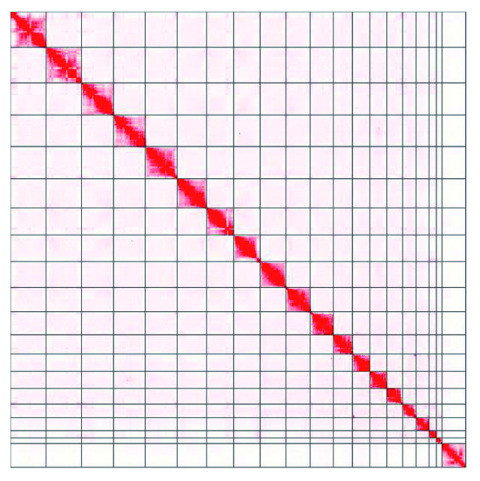
Genome assembly of *Sciurus vulgaris* mSciVul1: Hi-C contact map. Hi-C contact map of the *S. vulgaris* mSciVul1 assembly, visualized in Juicebox. The interactive version of this figure is available here, powered by Juicebox.js ( Robinson *et al.*, 2018).

**Table 2.** Chromosomal pseudomolecules in the genome assembly of *Sciurus vulgaris* mSciVul1.

ENA accession	Chromosome	Size (Mb)	GC%
LR738611.1	1	213.19	40.3
LR738612.1	2	204.37	40.9
LR738613.1	3	189.66	40.4
LR738614.1	4	187.75	39.4
LR738615.1	5	181.67	39.6
LR738616.1	6	173.03	39
LR738617.1	7	162.82	39.4
LR738618.1	8	153.87	40.6
LR738619.1	9	146.52	38.2
LR738620.1	10	145.29	38.9
LR738622.1	11	132.26	40.2
LR738623.1	12	115.30	40.2
LR738624.1	13	100.60	40.8
LR738625.1	14	99.24	41.3
LR738626.1	15	91.00	40.2
LR738627.1	16	79.70	42.9
LR738628.1	17	75.03	45.1
LR738629.1	18	41.58	47.9
LR738630.1	19	33.12	45.1
LR738621.1	X	138.34	37.9
LR738631.1	Y	4.04	38.9
-	unplaced	210.22	15.6

## Methods

The red squirrel specimen was collected from a garden in Beechwood Drive, Formby, Merseyside, L37 2DQ. Grid ref: SD2829706400 (Lat Long: 53.549316, -3.0836773) by the Wildlife Trust for Lancashire, Manchester and North Merseyside as part of an ongoing programme of recovery of dead squirrels. The spleen was dissected out during autopsy. A full tissue dissection and preservation in 80% ethanol was undertaken and the specimen accessioned by the Natural History Museum, London.

DNA was extracted using an agarose plug extraction from spleen tissue following the Bionano Prep Animal Tissue DNA Isolation Soft Tissue Protocol ^2^. Pacific Biosciences CLR long read and 10X Genomics read cloud sequencing libraries were constructed according to the manufacturers’ instructions. Sequencing was performed by the Scientific Operations core at the Wellcome Sanger Institute on Pacific Biosciences SEQUEL I and Illumina HiSeq X instruments. Hi-C data were generated by the Aiden lab using an optimised version of their protocols ( Dudchenko *et al*., 2017). BioNano data were generated in the Rockefeller University Vertebrate Genome laboratory using the Saphyr instrument. Ultra-high molecular weight DNA was extracted using the Bionano Prep Animal Tissue DNA Isolation Soft Tissue Protocol and assessed by pulsed field gel and Qubit 2 fluorimetry. DNA was labeled for Bionano Genomics optical mapping following the Bionano Prep Direct Label and Stain (DLS) Protocol and run on one Saphyr instrument chip flowcell. The total yield of tagged molecules ≥ 150 kb with at least 9 sites was 320.6 Gb (N50 0.25 Mb). A CMAP (Bionano assembly consensus genome map) was *de-novo* assembled using Bionano Solve (see Table 3 for software versions and sources) yielding 574 maps with a total map length of 3.28 Gb and a map N50 of 86.34 Mb.

**Table 3.** Software tools used.

Software tool	Version	Source
BioNano Solve	3.3	http://www.bnxinstall.com/solve/BionanoSolveInstall.html
Falcon-unzip	falcon-kit 1.1.1	Chin *et al.*, 2016
purge_dups	1.0.0	*Guan *et al.*, 2019*
scaff10x	4.2	https://github.com/wtsi-hpag/Scaff10X
3D-DNA	180419	Dudchenko *et al.*, 2017
Juicebox Assembly Tools	1.9.8	Dudchenko *et al.*, 2018
arrow	GenomicConsensus 2.3.3	https://github.com/PacificBiosciences/GenomicConsensus
longranger align	2.2.2	https://support.10xgenomics.com/genome-exome/software/pipelines/latest/ advanced/other-pipelines
freebayes	v1.1.0-3-g961e5f3	Garrison & Marth, 2012
bcftools consensus	1.9	http://samtools.github.io/bcftools/bcftools.html
gEVAL	2016	Chow *et al.*, 2016
BlobToolKit	1	Challis *et al.*, 2019

Assembly followed a modified version of the Vertebrate Genomes Project assembly protocols ^3^. In brief, assembly was carried out using Falcon-unzip ( Chin *et al*., 2016), haplotypic duplication was identified and removed with purge_dups ( Guan *et al*., 2019) and a first round of scaffolding carried out with 10X Genomics read clouds using scaff10x. Hybrid scaffolding was performed using the BioNano DLE-1 data and BioNano Solve.

Scaffolding with Hi-C data ( Rao *et al*., 2014) was carried out with 3D-DNA ( Dudchenko *et al*., 2017), followed by manual curation with Juicebox Assembly Tools ( Dudchenko *et al*., 2018; Durand *et al*., 2016; Robinson *et al*., 2018). The Hi-C scaffolded assembly was polished using arrow with the PacBio data, then polished with the 10X Genomics Illumina data by aligning to the assembly with longranger align, calling variants with freebayes ( Garrison & Marth, 2012) and applying homozygous non-reference edits using bcftools consensus. Two rounds of the Illumina polishing were applied. The assembly was checked for contamination and further manually assessed and corrected using the gEVAL system ( Chow *et al*., 2016). The genome was analysed within the BlobToolKit environment ( Challis *et al*., 2019).

## Data availability

### Underlying data

European Nucleotide Archive: Sciurus vulgaris (red squirrel) genome assembly, mSciVul1. BioProject accession number PRJEB35381; https://identifiers.org/ena.embl:PRJEB35381.

The genome sequence is released openly for reuse. The *S. vulgaris* genome sequencing initiative is part of the Wellcome Sanger Institute’s “25 genomes for 25 years” project ^4^. It is also part of the Vertebrate Genomes Project (VGP) ^5^ ordinal references programme, the DNA Zoo Project ^6^ and the Darwin Tree of Life (DToL) project ^7^. The specimen has been preserved in ethanol and deposited with the Natural History Museum, London under registration number NHMUK ZD 2019.213, where it will remain accessible to the research community for posterity. All raw sequence data and the assembly have been deposited in the ENA. The genome will be annotated and presented through the Ensembl pipeline at the European Bioinformatics Institute. Raw data and assembly accession identifiers are reported in Table 1.

## Footnotes

^1^ https://www.forestresearch.gov.uk/documents/667/fcpn5.pdf;

^2^ https://bionanogenomics.com/wp-content/uploads/2018/02/30077-Bionano-Prep-Animal-Tissue-DNA-Isolation-Soft-Tissue-Protocol.pdf

^3^ https://github.com/VGP/vgp-tools

^4^ https://www.sanger.ac.uk/science/collaboration/25-genomes-25-years

^5^ https://vertebrategenomesproject.org/

^6^ https://www.dnazoo.org/

^7^ https://www.darwintreeoflife.org/
