# Supplementary material for: The genome sequence of the Eurasian red squirrel, Sciurus vulgaris Linnaeus 1758
Source: Wellcome Open Res. 2020 Feb 3;5:18. [Version 1] doi: 10.12688/wellcomeopenres.15679.1 (PMC7309416; doi:10.12688/wellcomeopenres.15679.1)
Supplement: Peer review history

# Peer review: Reviewer response for version 1

**DOI:** 10.21956/wellcomeopenres.17184.r38866

**Published:** 2020-06-19

**Authors:** Rob Ogden

**Affiliations:** 1 Royal (Dick) School of Veterinary Studies and the Roslin Institute, University of Edinburgh, Edinburgh, UK

The article details the production and release of the first genome sequence for the Eurasian red squirrel, *Sciurus vulgaris*, generated from an individual squirrel sample collected in the United Kingdom. It provides a comprehensive explanation of the sequencing and assembly methods used, and resulting data availability, alongside a summary of key genome assembly characteristics. The release of the genome will provide an important reference sequence resource for future studies of red squirrel biology, in particular, investigations of immunogenetic diversity and population genetic research to support conservation management.

Are sufficient details of methods and materials provided to allow replication by others?

Yes

Is the rationale for creating the dataset(s) clearly described?

Yes

Are the datasets clearly presented in a useable and accessible format?

Yes

Are the protocols appropriate and is the work technically sound?

Yes

Reviewer Expertise:

Evolutionary and population genetics, applied conservation genetics.

I confirm that I have read this submission and believe that I have an appropriate level of expertise to confirm that it is of an acceptable scientific standard.

# Peer review: Reviewer response for version 1

**DOI:** 10.21956/wellcomeopenres.17184.r37784

**Published:** 2020-02-18

**Authors:** Peter H. Sudmant

**Affiliations:** 1 Department of Integrative Biology, University of California, Berkeley, Berkeley, CA, USA

In this manuscript Mead and colleagues report on a draft assembly of the Eurasian red squirrel, *Sciurus vulgaris*. The genome assembly is constructed from a combination of Pac-bio and 10X, HiC, and Bionano optical mapping, using a slightly modified standard VGP assembly protocols. The resulting assembly is largely assigned to 21 chromosomes with high scaffold N50 (~150Mb). Overall the manuscript concisely describes the genome and the presented resources will be of great use for population genetics on this species and related taxa. Some additional minor details on the assembly quality would be informative. 

**Specific comments:**

- Mention of the contig N50 would be helpful in addition to the number of gaps. 
- I cannot find any information about the base-pair quality of this reference? That too would be helpful.
- Citing the accompanying grey squirrel manuscript ^1^ would be useful, particularly as this genome is referred to in Figure 3.

Are sufficient details of methods and materials provided to allow replication by others?

Yes

Is the rationale for creating the dataset(s) clearly described?

Yes

Are the datasets clearly presented in a useable and accessible format?

Yes

Are the protocols appropriate and is the work technically sound?

Yes

Reviewer Expertise:

Genetics and genomics.

I confirm that I have read this submission and believe that I have an appropriate level of expertise to confirm that it is of an acceptable scientific standard.

## References

[1] : The genome sequence of the eastern grey squirrel, Sciurus carolinensis Gmelin, 1788. *Wellcome Open Research*.2020;5: 10.12688/wellcomeopenres.15721.1 10.12688/wellcomeopenres.15721.1 33215047PMC7653645
